# A hierarchy of needs? Embryo donation, *in vitro* fertilisation and the provision of infertility counselling

**DOI:** 10.1016/j.pec.2010.09.014

**Published:** 2011-11

**Authors:** Laura Machin

**Affiliations:** Science and Technology Studies Unit, University of York, UK

**Keywords:** Assisted conception clinics, Assisted conception techniques, Embryo donation, Infertility counselling, *In vitro* fertilisation

## Abstract

**Objective:**

The aim of the paper is to examine how those working in, using and regulating assisted conception clinics discussed infertility counselling and its provision within the context of embryo donation and *in vitro* fertilisation.

**Method:**

35 participants were recruited for semi-structured, face-to-face interviews. All data were analysed using thematic analysis.

**Results:**

The thematic analysis revealed recurring themes based upon the portrayals of infertility counselling, embryo donation and *in vitro* fertilisation.

**Conclusions:**

This paper suggests that an implicit hierarchy exists around those using assisted conception techniques and their infertility counselling requirements, which was dependent upon the assisted conception technique used. As a result, some people using assisted conception techniques felt that their needs had been overlooked due to this covert hierarchy.

**Practice implications:**

Those working in, using or regulating assisted conception clinics should not view infertility counselling as restricted to treatments involving donation, or solely for people within the clinical system.

## 1 Introduction

According to the Human Fertilisation and Embryology Authority (HFEA), the United Kingdom's (UK) regulatory body for the 114 licensed assisted conception clinics, approximately 3.5 million people in the UK will experience fertility problems across their lifetime [1]. Assisted conception techniques are the principal means of treating fertility problems. Yet, some techniques are used more frequently than others. In the UK, nearly 37,000 people used techniques involving their own gametes during 2007 compared to under 2000 people who used donated gametes and embryos to conceive [2]. Historically, the focus of infertility counselling provision has been upon the latter group.

It is well documented that assisted conception techniques can cause distress for those using them [3–9]. However, what is often forgotten is the distress can continue long after the treatment has been carried out, a pregnancy conceived or a baby born [7]. Counselling has received recognition for its role in the assisted conception technique process [4,10–13] and those using the techniques are thought to be in favour of counselling [5,14,15]. Yet, during recent Parliamentary discussions, it was suggested that the utilization of counselling can vary between 2% and 40% [16], which concurs with studies conducted in clinics in the UK [17]. A number of factors are thought to influence whether people access counselling or not, such as the timing of it [3,14,18,19]; the perception of the techniques [20,21]; and the relationship between clinicians and counsellors within clinics [16,22,23].

Studies in countries where infertility counselling is mandatory, such as parts of Australia, have explored the experiences of those using assisted conception techniques and have concluded that it is important to recognise that some people may not want or need counselling [7] in order to avoid defensive behaviour from those using the techniques [21]. In countries where infertility counselling is optional, such as Denmark, it has been considered whether the emotional needs of those using assisted conception techniques could be met by other clinical staff [15]. However, optional counselling has been thought to make those utilizing it feel inadequate in some way [10,23].

The matter of clinics offering counselling to those using assisted conception techniques arose with the introduction of IVF in 1978 [24]. The announcement of the first baby conceived from a donated embryo in 1983 [25], was to complicate counselling provision. During this time, recommendations from various bodies were put forward regarding the provision of counselling [26], but it would not be until the passing of the Human Fertilisation and Embryology (HFE) Act (1990), that it would become formalised.

The Act established the HFEA who produces a Code of Practice, which makes reference to counselling. All UK clinics have to abide by the 1990 Act, which was amended by the 2008 HFE Act. Currently, clinics in the UK are obligated to *offer* counselling to every person using assisted conception techniques, but are required to offer *additional* counselling for those using donated embryos, eggs or sperm, in order to consider the implications arising from using donated material to conceive [27]. The issue of ‘offering’ counselling, rather than making it mandatory, was discussed during the amendments to the Act, with key stakeholders from patient groups, the counselling profession and academia, highlighting the difficulties that can arise with it e.g. the interpretation of the ‘offer’ of counselling varying between clinics [13].

It is the distinction between treatments that involve donated gametes or embryos or not, in the counselling provision, that is of most interest. How do those affected by the distinction i.e. those using, working or regulating assisted conception clinics, discuss counselling and assisted conception techniques? Embryo donation and IVF provide the perfect backdrop for this discussion as they are deemed to be technically associated with each other [28–30], as embryos created during IVF can be made available for donation. Consequently, by considering both IVF and embryo donation, a broader group of people are implicated, such as stem cell scientists, embryo donors for research purposes, as well as those with frozen embryos after completing treatment.

## 2 Methods

### 2.1 Participant characteristics

A total of 35 interviews were conducted between February 2006 and January 2007. Participants were categorised into three broad groups, based upon their association with assisted conception clinics: those who worked within clinics (16), those who used clinics (12) and those who were involved in the regulation of clinics (7). The intention was to gather a broad spectrum of voices of those involved in the IVF and embryo donation processes, such as clinicians, infertility counsellors, fertility nurses, clinical embryologists, stem cell scientists, people who had used IVF or donated embryos to conceive, people who had completed treatment and had frozen embryos stored, and people who donated their frozen embryos for research or treatment purposes.

Assisted conception clinics were identified where both embryo donation and IVF were conducted. As the statistics showed, embryo donation was rarely carried out in clinics, therefore the amount of participants that could be recruited was limited. Furthermore, not every clinic had a stem cell laboratory attached to it, nor did every clinic have counsellors on-site. It was not intended to collect a representative sample, and it was expected that a number of participants would be identified through recommendations from other participants once data collection began.

### 2.2 Procedure

Participants who worked in, or regulated, clinics where IVF and embryo donation were conducted, were recruited via email. Participants who had played a prominent role in the development of embryo donation or IVF were particularly approached due to the distinction made between the two techniques in the counselling provision.

People who had used assisted conception techniques were recruited through advertising on infertility support groups websites and newsletters. A number of support group representatives were interviewed and asked to pass details of the project on to people they knew had used assisted conception techniques. Recruiting people who had used assisted conception techniques through clinics was avoided as this would intensify the already lengthy ethical approval sought when interviewing other participants associated with hospitals and clinics.

Face-to-face, semi-structured interviews were conducted as they gave both the participant and the interviewer freedom and flexibility to follow up topics that might not initially have been on the interview guide [31,32]. It also ensured that the data collected were rich and in-depth. Each of the interviews were tape-recorded and lasted between 1 and 2 h. All interviews were transcribed at length.

### 2.3 Data analysis

A social constructionist approach [33] was adopted, which meant the data was read critically, and analysed and treated as accounts rather than simply taking them at face value. Social constructionists argue that reality is constructed through discourse and are primarily interested in both the production and meaning of reality [34,35]. As a result, the focus was upon the meaning-making process rather than gathering details on the beliefs, traditions and personality traits of the participants interviewed. The data therefore, were examined for what meanings were created and how they were constructed [33,36,37].

The analysis was an iterative process. The data were inputted into a qualitative computer package, Nvivo7, and coded for themes. The codes derived from initial research questions and were based upon very broad themes, such as the portrayal of embryo donation, IVF and counselling. On average, each transcript was read four times, with new codes emerging with each reading, such as portrayal of embryo donors and embryo recipients, or existing codes becoming more refined, such as portrayal of embryo donation for infertility purposes or for research purposes. Importantly, any ‘unexpected issues’ [38] that emerged during the reading of the data were also acknowledged, which resulted in further refinement of the codes. Finally, the codes were considered in light of the research questions and were brought together to illustrate a single conclusion or story [39].

To ensure the quality of the data analysis a number of approaches were adopted. The interviews were conducted over a period of time, thereby allowing for initial observations to be confirmed and test emerging analytical findings. Documentary data collected had a similar function. Analytical summaries and data transcripts were read by, and discussed with, colleagues. Whilst the analysis evolved in light of these discussions, and the documentary data and interviews collected, it did not significantly alter.

### 2.4 Ethical considerations

Ethical approval was obtained from 3 sources: the Faculty at the University of Leeds, the NHS Central Office for Research Ethics Committees and each individual National Health Service Trust, depending upon where the participant was based.

Each participant was sent an informed consent form via email before the interview, providing the opportunity to review and discuss it with other people if they wished, and was signed in front of the researcher. Each participant was asked if the interview could be tape-recorded and it was explained to them that the interview could be stopped at any point, that it was completely confidential and anonymous. To protect the privacy and confidentiality of the participants, all personal identifying details have been removed from this paper.

## 3 Results

Through the thematic analysis, recurring themes were identified, around the portrayals of counselling, embryo donation and IVF. Extracts of the data are used and are ordered according to the theme they illustrate, rather than the 3 interviewee groups. Although the author of the quote is referred to, it is hoped that by ordering the data according to themes, the nuances of the data are maintained.

### 3.1 Infertility counselling as ‘necessary’

All (35) participants stated that counselling was necessary for people who *used* donated gametes or embryos to conceive. 3 participants made specific reference to the amount of counselling that embryo and gamete recipients received, as this quote from an infertility support group representative illustrated,

…the vast majority of clinics won’t do gamete or embryo donation without people having at least one counselling session. What concerns me is that one isn’t enough, *particularly if you’re doing embryo donation*. (SG_02)

13 participants working in clinics implied that assisted conception techniques that involved ‘donation’ were more ‘problematic’ than IVF in some way, as a quote from an embryologist at a large NHS clinic demonstrated,

…if you have any donated genetic material you should always be offered and encouraged to take counselling because *that's a whole other step to having infertility treatment for yourselves*… (Emb_01).

In the context of embryo donation, counselling was portrayed positively and something that should be encouraged by those working in clinics. The clinical embryologist inferred that those working in clinics had a responsibility to promote counselling, albeit limited to those using donated embryos.

People who *donated* their embryos were also highlighted by 10 participants as requiring counselling. Yet, this was limited to those donating embryos for treatment purposes. When all (35) participants were asked about counselling for those donating embryos, 30 answered this question in relation to embryos donated to others to be used for conception. People donating their stored embryos to stem cell research were rarely considered by those working in clinics as requiring counselling.

However, a consequence of portraying counselling as necessary for those using donated embryos to conceive, was it became viewed by those having treatment as an obstacle to overcome. A woman who had conceived her daughter using donated embryos explained how counselling was something she had to do in order for her treatment to progress,

I don’t wish we had more counselling before I was pregnant because if they’d said to me you’ve got to abseil down that cliff and up that wall…I would have done whatever they said I needed to do. We only really did that counselling session because we had to. (ER_Pat_03)

The above quote raises doubts over how valuable the counselling was perceived by those using assisted conception techniques when a compulsory policy was adopted.

People using assisted conception techniques that did *not* involve donated gametes or embryos were not considered by those working in or regulating clinics as requiring similar encouragement to access counselling. In discussing IVF in the context of embryo donation, 12 participants who worked in clinics argued *against* counselling for people using their own gametes in treatment. A stem cell scientist referred to the technical simplicity of the IVF process to claim counselling was unnecessary,

…IVF couples shouldn’t be exposed to *too much scrutiny* or invasive procedures simply because they need an IVF lab to put their sperm and eggs together, which is essentially all it is in simple cases…(Sci_03)

Embryo donation was portrayed as more complex than IVF. As such, an association between counselling and ‘problematic’ treatments was formed. An infertility counsellor at a large NHS clinic explained the clinic's policy for counselling for embryo donation and IVF programmes,

…we do about 500 [counselling] sessions a year seeing everybody who's going to use any donor gametes…And then anybody who have problems with fertility or investigations or treatment, *we see if they want to be seen*. (Coun_01)

From the above quote, it was possible to conclude that the onus was placed upon those using assisted conception techniques that did not involve donated gametes or embryos to request counselling, although for some participants, this proved a difficult task.

### 3.2 Gaining access

5 participants who had used IVF implied that counselling was inaccessible and something they felt unable to ask for. A woman who had taken a break from treatment after experiencing two failed cycles of IVF, referred to the optional nature of the current policy of counselling for all patients and the difficulties it presented as a patient,

[if] they made you see someone then you wouldn’t have a choice and you would be fine about it. It's making that step to see a counsellor is actually quite hard. I think once you’ve got there you’re not so bad, but saying I’m struggling here, which surely to God everybody struggles?…so everybody should have had counselling. It's [counselling policy] just so wishy-washy and it's [counselling] there if you need it…(IVF_Pat_01)

The same participant also summarised how those who used assisted conception techniques often reported looking to fertility nurses, clinicians or clinical embryologists for a referral to counselling,

…all you wanted was for somebody just to say one thing and you would have broke. Because you’re in the middle of it all, you’re stressed to the hilt…There would have been tears and hopefully they would have then referred me to a counsellor…But never. It's just quickly rush you through the clinic…no real person time and it's [IVF] such a sensitive issue. (IVF_Pat_01)

When discussing the difficulty in requesting a referral for counselling, 3 participants who had used assisted conception techniques explained how they accessed other forms of ‘counselling’ external to the clinics. A woman who had conceived her daughter on her first cycle of IVF explained how she approached her general practitioner during her treatment in order to receive some ‘support’,

I did go along to my GP and had a few tears in the surgery…saying I just don’t think I’m coping with this [IVF] very well. And he said, well how do you think you should be coping because this is a big deal? I said, well I don’t know but I just think I should be coping better and I just feel I need some help to cope…(IVF_Pat_04)

However, 9 participants working in clinics assumed that those using assisted conception techniques, such as IVF, would ask for, or refer themselves to, counselling if they felt they needed it. This highlighted a disparity between those using, and those working in, clinics and their perceptions of access to counselling.

### 3.3 Dealing with the aftermath

11 participants working in, and 4 regulating, clinics were unaware of the difficulties that people had after they had completed treatment and were outside of the clinical system. Regulators (4), clinicians (5) and clinical embryologists (3) presented IVF as ‘normalised’ and accepted within society. Conversely, 5 participants who had used IVF reported struggling to tell their parents, their children and their friends about their treatment, and of their decision to donate embryos. It was the decision around frozen embryos that caused most distress, as a woman who conceived her daughter through IVF explained. When interviewed, the time limit for her stored embryos was approaching and she felt her needs arising from this matter had been overlooked by the clinic where she had her treatment,

…other than sending us a form every year saying do you wish storage to continue…there is no other form of support. And I did feel very, very sort of dropped…I think people are losing sight of the fact that there are some big issues that crop up [with IVF] and that they should have some support. (IVF_Pat_04)

However, 4 infertility counsellors recognised that making the decision over stored embryos a time when they were most required by people who had used assisted conception techniques, as the following quote highlighted,

…some people do see embryos as babies…They’ll come to counselling because they don’t want to dispose because they don’t want to kill the embryos. They don’t want to have it hacked up in a laboratory somewhere. They don’t want to give their baby away to somebody else. They don’t want anymore children. So they’re stuck. (Coun_03)

Interestingly, counselling was portrayed by 5 participants using assisted conception techniques and 4 infertility counsellors as ‘support’, particularly when conducted *after* treatment.

## 4 Discussion and conclusion

### 4.1 Discussion

The purpose of the present study was to qualitatively examine how those working in, using, and regulating assisted conception clinics discussed counselling in the context of IVF and embryo donation. From the data, it appeared that the *type* of assisted conception technique used influenced how counselling was discussed. Similarly, the perception of counselling influenced how particular assisted conception techniques, such as IVF and embryo donation, were discussed.

It was possible to discern that a hierarchy around those using assisted conception techniques and their counselling requirements existed implicitly within the data. The hierarchy was dependent upon the technique used. At the top of the hierarchy were people using donated embryos to conceive. They were deemed by all (35) participants as *requiring* counselling, *before* beginning treatment and on *numerous* occasions. Those using donated eggs or sperm to conceive were also deemed in need of counselling, again before treatment commenced. Yet, 8 participants suggested that the ‘need’ for counselling was not as great as those using donated embryos, therefore positioning those using donated gametes as lower down the hierarchy.

People donating embryos were also considered by 10 participants, usually counsellors, as requiring counselling, although two subtle distinctions were apparent with this group of people. Firstly, the direction of the donation i.e. to stem cell research or to other couples, appeared to influence whether participants considered counselling as necessary. Secondly, participants distinguished between those making the decision around their stored embryos and those who had opted to donate their frozen embryos. In particular, counsellors pinpointed both making the decision and donating embryos as significant situations requiring counselling, but it was the role they could play in the decision-making around frozen embryos that they emphasised most. This is perhaps not surprising given that those with frozen embryos still had a ‘connection’ with the clinic and therefore not totally outside of the clinical system, whilst those who opted to donate their embryos arguably severed this ‘connection’. Therefore the next level of the hierarchy in terms of counselling requirements were *both* those that donate their embryos to other couples and those who were making decisions over their frozen embryos.

Beneath these in the hierarchy were those who donated their embryos to stem cell research as they were rarely regarded by those working in clinics as requiring counselling. Further research is required in order to explore how embryo donation for stem cell research has been perceived by those working within clinics and how this relates to embryo donors’ experiences of treatment. Finally, those that used IVF were considered by 12 participants working in clinics as at the bottom of the hierarchy in terms of their counselling requirements.

Previous studies on the utilization of counselling by those using assisted conception techniques, concluded that clinics needed to be proactive in identifying those who need support [21] and counselling should be ongoing [7]. Similarly, 7 participants in this study who had used assisted conception techniques claimed that counselling was necessary *regardless* of the technique used, and something that should be promoted by those working within assisted conception clinics, *throughout* the treatment process. For those using assisted conception techniques, the treatment ‘journey’ [7] did not end once a baby was conceived or born, but instead, brought new matters that required assistance. The 7 participants in this study positioned counselling as a way of dealing with the unintended consequences from treatments.

The disparities in the hierarchy between those using and working within clinics highlighted the disjointedness between the provision and need for counselling. When contemplating the figures available from the HFEA for the use of assisted conception techniques during 2007, only 2000 people were considered as having ‘necessary’ counselling requirements, in relation to the hierarchy found in this study. The findings arising from this study suggest that those using assisted conception techniques felt that their needs were being overlooked.

Discussing counselling provision in such a way raises the debate around making counselling mandatory, either for specific groups of people or for all people using clinics. Obviously, there is a cost issue attached to these options, although this does not belie the responsibility that regulators and clinics arguably have towards those using assisted conception techniques. Furthermore, it has been shown here and elsewhere [15], that not every person having treatment feels they need counselling, although they can still feel reassured and helped by its availability and utilization [10]. A way to overcome both of these issues would be for the culture surrounding counselling to shift, specifically within clinics. It has been recognised that nurses and general practitioners can play a role in offering support to those using assisted conception techniques [7]. However, others have claimed it is too much to ask of clinical staff, given the other demands associated with their roles. Furthermore, those using assisted conception techniques can be left feeling that their needs are overlooked if reliant solely upon clinical staff for support [15]. The findings from this study have shown that the role of the clinical team i.e. clinician, fertility nurse, clinical embryologist, in influencing counselling utilization, should not be underestimated. To assist this process, the role and contribution of the counsellor should be valued and understood by the clinical team as suggested by Monach [23]. Finally, those within clinics need to recognise counselling as having the *potential* to be for everyone, rather than treatment-specific, and something that needs to be encouraged *throughout* the treatment process.

### 4.2 Conclusion

This paper suggests that an implicit hierarchy exists around those using assisted conception techniques and their counselling requirements, which was dependent upon the technique used. As a result, some people using assisted conception techniques felt that their needs had been overlooked, due to this covert hierarchy.

### 4.3 Practice implications

Those working in, using or regulating clinics should not view counselling as restricted to treatments involving donation, or solely for people within the clinical system.

I confirm all patient/personal identifiers have been removed or disguised so the patient/person(s) described are not identifiable and cannot be identified through the details of the story.
